# Supplementary material for: Control of electronic band profiles through depletion layer engineering in core–shell nanocrystals
Source: Nat Commun. 2022 Jan 27;13:537. doi: 10.1038/s41467-022-28140-y (PMC8795196; doi:10.1038/s41467-022-28140-y)
Supplement: Supplementary file 1 — Supplementary Information [file 41467_2022_28140_MOESM1_ESM.pdf]

Supplementary Information

Control of electronic band profiles through depletion layer engineering in core-shell nanocrystals

Michele Ghini[1,2,3], Nicola Curreli[1]*, Matteo B. Lodi[4], Nicolò Petrini[1,5], Mengjiao Wang[1,6], Mirko Prato[6], Alessandro Fanti[4], Liberato Manna[2], Ilka Kriegel[1]*

[1] Functional Nanosystems, Istituto Italiano di Tecnologia, via Morego 30, 16163 Genova, Italy
[2] Nanochemistry Department, Istituto Italiano di Tecnologia, via Morego 30, 16163 Genova, Italy
[3] Dipartimento di Chimica e Chimica Industriale, Università degli Studi di Genova, Via Dodecaneso 31, 16146 Genova, Italy
[4] Department of Electrical and Electronic Engineering, University of Cagliari, via Marengo 2, 09123, Cagliari, Italy
[5] Dipartimento di Fisica, Università degli Studi di Genova, Via Dodecaneso 33, 16146 Genova, Italy
[6] Materials Characterization Facility, Istituto Italiano di Tecnologia, Via Morego 30, 16163, Genova, Italy
These authors contributed equally: Michele Ghini, Nicola Curreli.
*email: nicola.curreli@iit.it; ilka.kriegel@iit.it

Supplementary Figures

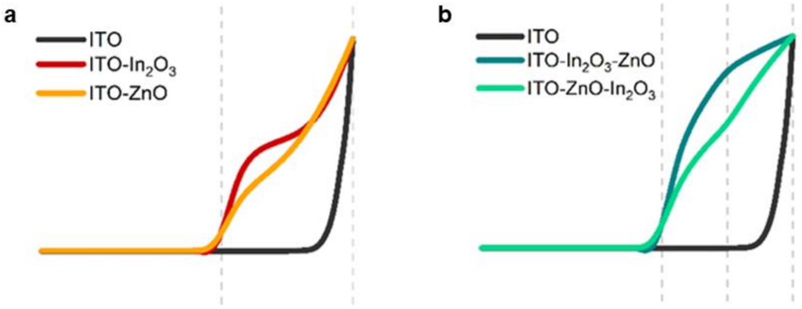

Supplementary Figure 1. **Depletion layer engineering of metal oxide NCs via tuning of structural and electrical properties.** Effects of multiple shells of different materials surrounding an ITO core radius of 5.5 nm. A homogeneous ITO NCs of the same total radius (total radius = 9.5 nm) is shown in black, as comparison. **a** ITO-In_2O_3 core-shell and ITO-ZnO core-shell NCs. **b** ITO-In_2O_3-ZnO core-multishell and ITO-ZnO-In_2O_3 core-multishell NCs. Each shell has a thickness of 2 nm, for a total NC radius of 9.5 nm.

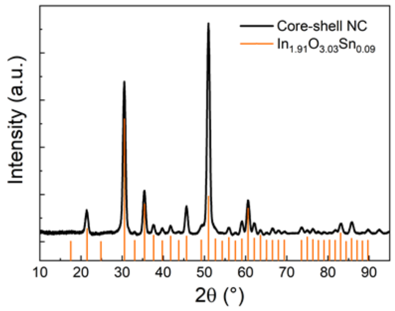

Supplementary Figure 2. **XRD of the ITO-In_2O_3 core-shell NCs**, in agreement with the 98-005-0847 card for ITO.

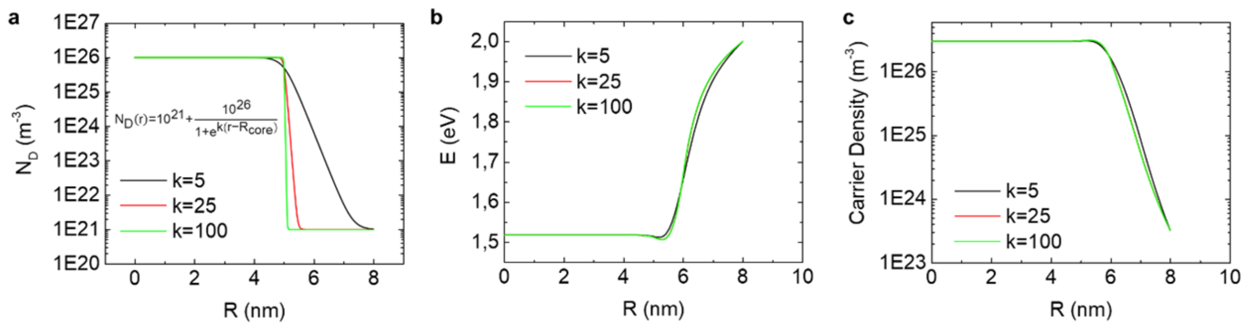

Supplementary Figure 3. **Simulated effects of dopants diffusion.** By varying the shape factor ($k = 5, 25, 100$), we induce a variation of the dopant distribution within the core-shell nanocrystal. **a** The Poisson's equation was solved, and the band diagram and electron density were analysed finding that differences in the conduction band and electron density profiles are negligible. **b** The numerically calculated energy levels. **c** Numerically calculated carrier density profiles of an ITO-In_2O_3 core-shell NC with the donor profiles input as shown in panel a.

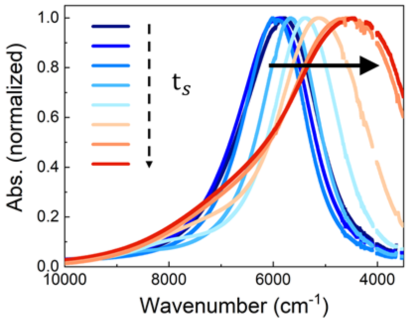

Supplementary Figure 4. **Normalized absorption spectra of ITO-In_2O_3 core-shell samples.** By growing a subnanometric shell, the peak position of the LPSR gets blueshifted, due to the activation of dopants at the surface of the ITO core. Further growth of undoped shell has the effect of redshifting the main peak of the LSPR in the core-shell system.

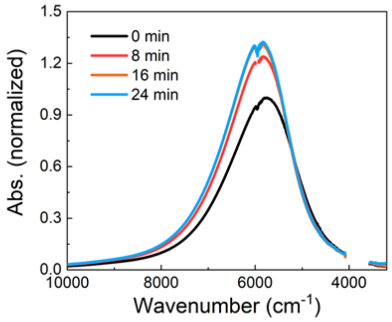

Supplementary Figure 5. **Photodoping ITO-In_2O_3 core-shell NCs.** Temporal evolution ($t = 0, 8, 16, 24$ min of UV exposure) of the LSPR absorption lineshape during the photodoping process. Most of the variations occurs in the first minutes, as expected for the charging of a capacitor-like system. After 16 minutes of UV exposure, photodoping saturates, with little modification of the LSPR lineshape.

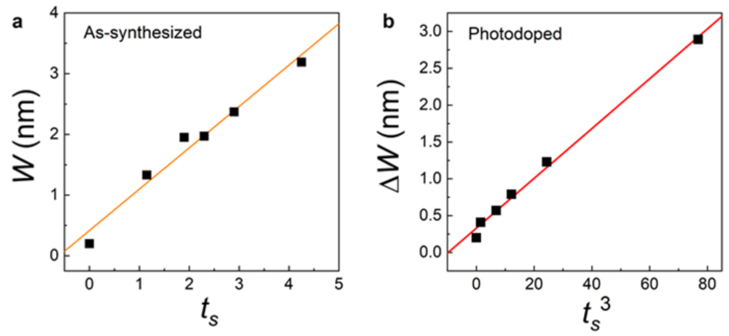

Supplementary Figure 6. **Dependence of main parameters on the shell thickness. a** Depletion layer width as a function of the shell thickness t_s. A linear fit is reported in orange. **b** The contraction of the depletion layer width (ΔW) in core-shell ITO-In_2O_3 NCs follows a t_s^3 law (linear fit showed in red), with t_s being the shell thickness.

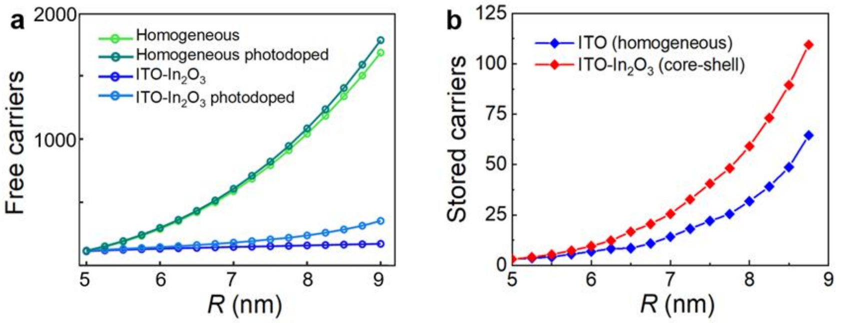

Supplementary Figure 7. **Comparison between the core-shell architecture and homogeneous particles. a** Numerical calculations of the total number of free carriers in the two cases of a homogeneous NC (ITO – core only) and core-shell NC (ITO-In2O_3), as a function of the shell thickness before and after photodoping. **b** Number of photocarriers stored in the two cases as a function of the radius.

Supplementary Notes

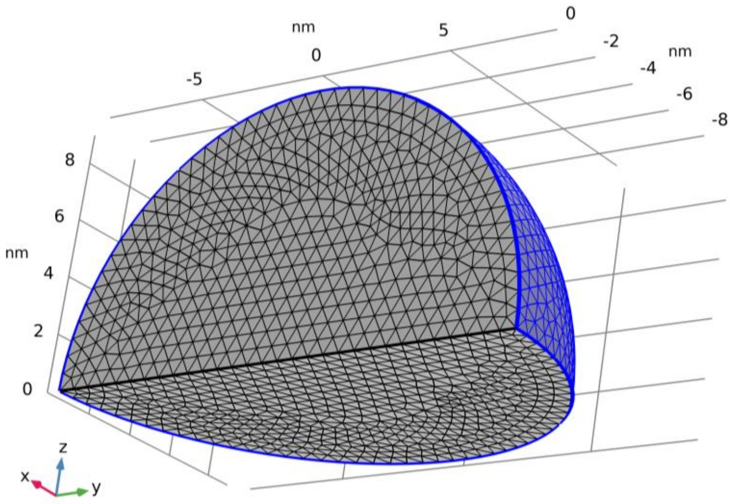

Supplementary Figure 8. **Meshed geometry of a spherical nanocrystal.** Poisson's equation was solved over this control volume using a finite element method in COMSOL.

For a spherical nanocrystal (Supplementary Figure 8) with given radial dopant profile, N_D and surface potential, E_S, we solved numerically the Poisson's equation using a finite element method (FEM)

software Comsol Multiphysics v5.6 (Comsol Inc., Burlinghton MA USA) by using the built-in *Mathematics module* exploiting the central symmetry of the sphere as shown in Supplementary Figure 8.

We adapted the dimensionless form of Poisson's equation in Cartesian coordinates derived by Seiwatz and Green:[1–3]

$$\nabla^2 u = -\frac{\rho \cdot e^2}{\varepsilon \varepsilon_0 k_B T} \quad (1)$$

where e is the electron charge, k_B is the Boltzmann constant, T is temperature, ε_0 is the vacuum permittivity, ε is the static dielectric constant, and ρ is the charge density. The non-dimensional potential u refers to the difference between the neutral bulk and the surface potentials. It is defined as:[1]

$$u = u_{bulk} - u_{surface} = \frac{E_F - E_I}{k_B T} \quad (2)$$

where E_F is the Fermi energy level, and $E_I = \frac{E_{CB} + E_{VB}}{2}$ is the reference potential and center of the band gap in which E_{CB} and E_{VB} are the conduction band and valence band profile respectively.

It is possible to expand the Poisson's equation by defining the charge density, $\rho = \rho(r)$ given by the following relation:

$$\rho = \rho_D(r) + p - n \quad (3)$$

where $\rho_D(r)$ is the radially changing donor dopant density, while p and n are the contribution of the hole and electron density, respectively.

Using an auxiliary function $w_{x,y} = \frac{E_x - E_y}{k_B T}$ it is possible to further define the terms of the charge density. Given the donor energy level, E_D, the activated dopant concentration can be expressed as:

$$\rho_D(r) = \frac{N_D(r)}{1 + 2e^{(u - w_{D,I}(r))}} \quad (4)$$

where $w_{D,I} = \frac{E_D - E_I}{k_B T}$.

The free hole (p) and electron (n) concentration in the parabolic conduction band are equal to:

$$p = 4\pi \left[\frac{2 m_h k_B T}{h^2}\right]^{3/2} F_{1/2}\big(w_{V,I}(r) - u\big);$$

$$n = 4\pi \left[\frac{2 m_e k_B T}{h^2}\right]^{3/2} F_{1/2}\big(u - w_{C,I}(r)\big) \quad (5)$$

where $w_{V,I} = \frac{E_{VB} - E_I}{k_B T}$, $w_{C,I} = \frac{E_{CB} - E_I}{k_B T}$ and h is the Planck's constant.

Here, the Fermi-Dirac distribution is assumed for the carriers:

$$F_{\frac{1}{2}}(\eta) = \int_0^{\infty} \frac{x^{1/2} dx}{1 + e^{x - \eta}} \quad (6)$$

being x the dummy variable, and the upper integration limit is set to ∞ on account of the fact that the integrand vanishes exponentially at high energies. From a numerical point of view, the upper bound of the integral was selected to be a finite number high enough to ensure the convergence to a solution, and to retain an accuracy within the 1%.

The Poisson's equation become:

$$\nabla^2 u = -\frac{e^2}{\varepsilon\varepsilon_0 k_B T}\left\{\frac{N_D(r)}{1+2e^{(u-w_{D,I}(r))}} + 4\pi\left[\frac{2m_h k_B T}{h^2}\right]^{3/2} F_{1/2}\left(w_{V,I}(r)-u\right) - 4\pi\left[\frac{2m_e k_B T}{h^2}\right]^{3/2} F_{1/2}\left(u - w_{C,I}(r)\right)\right\} \quad (7)$$

where m_h is the effective hole mass equal to 0.6 times the electron mass and m_e is the effective electron mass equal to 0.4 times the electron mass.

Parameters used in the simulations

Supplementary Table 1. **Parameters used in the simulations.**

e	ε	k_B	T	m_h	m_e	h
$1.602 \cdot 10^{-19}$ C	$8.854 \cdot 10^{-12}$ Fm^{-1}	$1.381 \cdot 10^{-23}$ JK^{-1}	293.15 K	$5.466 \cdot 10^{-31}$ kg	$3.644 \cdot 10^{-31}$ kg	$6.626 \cdot 10^{-34}$ Js

For the simulations, we used the parameters introduced in the Supplementary Table 1. In the case of nanocrystals made of only one material, energy levels, number of donors and relative dielectric permittivity are constant.

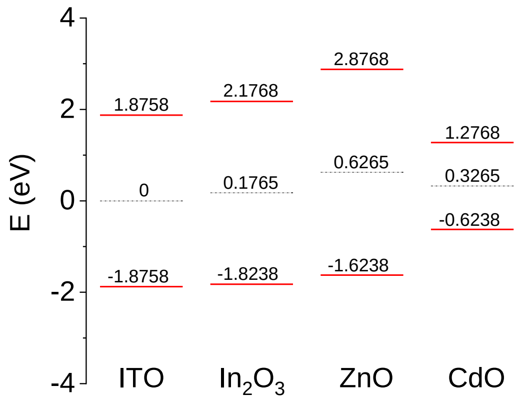

Supplementary Figure 9. **Relative conduction and valence band levels for ITO, In_2O_3, ZnO and CdO nanocrystals.** The energy levels are referred as non-equilibrium states. Dashed lines represent the intrinsic level.

For systems made of combination of different materials, since the NCs are material-dependent, we must define the radius-dependent non-equilibrium potentials profiles, donor distribution profile ($N_D = N_D(r)$) and relative dielectric profile ($\varepsilon(r)$).

For example, in the case of ITO-In_2O_3 nanocrystals, the radius-dependent non-equilibrium potentials profiles and donor distribution profile used to solve the Poisson's equation are shown in Supplementary Figure 10.[4] Different distributions can be found for ZnO and CdO (Supplementary Figure 9).[5–7]

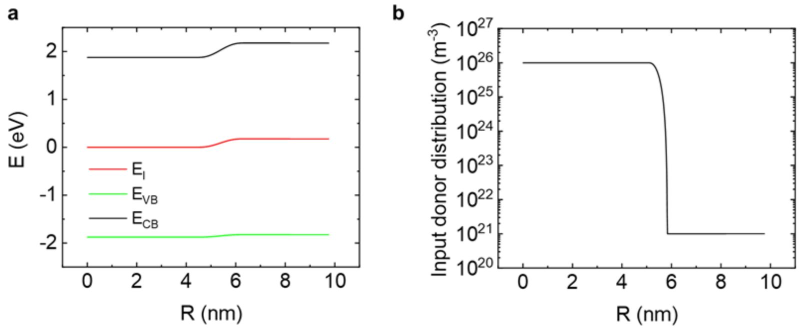

Supplementary Figure 10. **Potential and donor distribution in ITO-In_2O_3 nanocrystal with 6 nm core and a 5 nm shell**. **a** Input band diagram for a core-shell ITO- In_2O_3 structure. The system reference is the intrinsic level of the core material. **b** Input donor distribution as a function of the nanocrystal radius.

The diffusion effects on the NCs are investigated by modifying the donor distribution profile $N_D(r)$ (Supplementary Figure 3). In detail, we used a sigmoid-like function to model the diffusion effects:

$$N_D(r) = 10^{21} + \frac{10^{26}}{1+e^{k(r-r_{core})}} \tag{8}$$

By varying the shape factor ($k = 5, 25, 100$), the Poisson's equation was solved, and the band diagram and electron density were analysed again finding that differences in the conduction band and electron density profiles are negligible.

For each system, the energy levels and carrier density profiles after equilibrium were obtained according to the specific material taken into account and their combinations. The results can be seen in Figure 1 of the main text and in Supplementary Figure 1.

Photodoping

To study the core-shell system after photodoping we introduced additional generation and recombination terms into the Poisson's equation:[8]

$$\nabla^2 u = -\frac{\rho \cdot e^2}{\varepsilon \varepsilon_0 k_B T} + G(r) - R(r) \tag{9}$$

The recombination effects are neglected, hence $R(r) = 0$. In particular, the generation term $G(r)$ is spatially dependent and is modelled by the following Gaussian distribution:

$$G(r) = \frac{N_{max}}{V} \frac{1}{\sigma\sqrt{2\pi}} e^{\frac{r-R_{NC}}{\sigma}} \tag{10}$$

where N_{max} is the maximum and peak number of photoelectrons and σ is the shape factor which determine the spatial distribution of the photoelectrons into the nanocrystal. By varying σ we are able to model the effects on the energy levels after the photodoping and its effect on the band profiles. The results are shown in Figure 2 of the main text.

Electrons stored

The total number of electrons in the NC is calculated by integrating the electron density over the particle volume (V):

$$N_E = \int_V n(r)dV \quad (11)$$

The trend found by numerical simulations is in accordance with the experimental findings (Figure 4 main text). The simulation tends to underestimate the number of stored electrons for small t_s, however the error is within the margin of uncertainty of this method. We computed the number of stored electrons for all cases, and the results are shown in Figure 4 of the main text.

Optical simulations

In Supplementary Figure 11 and Figure 2d of the main text, we show the numerical simulations of absorption spectrum of ITO-In_2O_3 NCs. We couple the solution based on the radial carrier distribution obtained from the Poisson's equation, with the high-frequency formulation of the Maxwell's equation relating the electronic density of the NCs to the plasmonic resonance and simulating the absorption spectra. The simulations are carried out in Comsol by using the *Electromagnetic Waves, Frequency Domain module* adding a background medium box 10 times bigger than the nanocrystal radius to compute the far-field contribution.

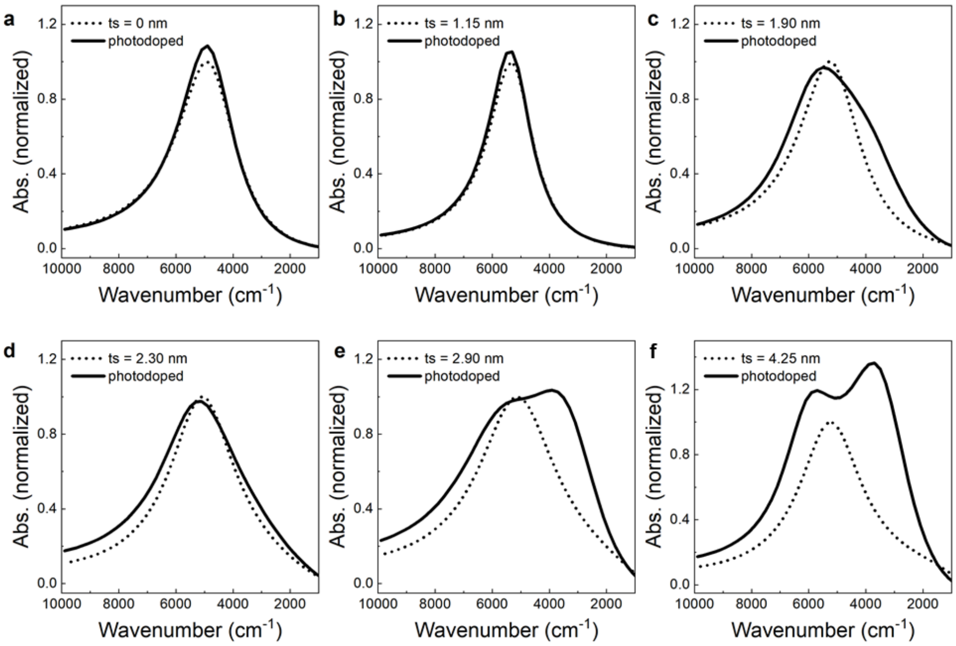

Supplementary Figure 11. **Numerical simulations of the absorbance of the ITO-In_2O_3 core-shell NC** before (dotted line) and the after the addition of extra electrons (*i.e.*, photodoping, solid line). Values of t_s were selected in order to match the samples C0-S5 we extensively analyzed. Each panel is representative of a different shell thickness (t_s). **a** t_s = 0 nm; **b** t_s = 1.15 nm; **c** t_s = 1.90 nm; **d** t_s = 2.30 nm; **e** t_s = 2.90 nm; **f** t_s = 4.25 nm.

Optical model

We estimate the number of electrons accumulated via photodoping from the experimental absorption spectra of ITO-In_2O_3 NCs by implementing an optical model based on Mie solution and effective medium approximation. Briefly, we considered Mie solution under the hypothesis of spherical particles in the quasi-static limit, and assuming a negligible retardation effect. Moreover, the effect of scattering is also negligible and can be disregarded. Under those conditions, the absorption relation can be written as:[9]

$$ABS = \frac{\sigma_{abs} NL}{ln(10)} \tag{12}$$

where σ_{abs} is the far-field absorption cross-section, N is the volume density of particles, L is the light pathlength through the cuvette. Considering the hypothesis of dominant dipolar mode,[10] absorption cross-section can be calculated as:

$$\sigma_{abs} = 4\pi k_m R^3 Im\left\{\frac{\varepsilon - \varepsilon_m}{\varepsilon + 2\varepsilon_m}\right\} \tag{13}$$

where $k_m = \frac{2\pi}{\lambda}\sqrt{\varepsilon_m}$ is the wavevector in the medium m, and R is the particle radius, ε is the dielectric permittivity of the nanoparticle and ε_m is the permittivity of the medium. For a homogeneous particle, the dielectric permittivity ε can be defined in the framework of the free-electron Drude-Lorentz model for bulk metals and doped semiconductor:

$$\varepsilon(\omega) = \varepsilon_\infty - \omega_P^2/(\omega^2 + i\omega\Gamma) \tag{14}$$

where ε_∞ is the high frequency dielectric permittivity, ω_p is the bulk plasma frequency and Γ is a damping parameter accounting for electron-electron scattering. In the case of ITO NCs, the damping parameter is described by an empirical frequency-dependent damping function:

$$\Gamma(\omega) = \Gamma_L - \frac{\Gamma_L - \Gamma_H}{\pi}\left[tan^{-1}\left(\frac{\Gamma_X}{\Gamma_W}\right) + \frac{\pi}{2}\right] \tag{15}$$

where Γ_L and Γ_H are the low and high frequency dumping constants, Γ_X is the crossover frequency in the mixed regime and Γ_W is the width of the crossover region. Frequency-dependent damping function accounts the electrons scattering of ionized impurities and allows accurately reproducing the asymmetry of the plasmonic resonances of ITO NCs.[11,12]

In the Equation (14), the bulk plasma frequency, ω_p is a function of the free carrier density (n_e) and the effective electron mass (m^*):

$$\omega_p = \sqrt{\frac{n_e e^2}{\varepsilon_0 m^*}} \tag{16}$$

where e is the electron charge and ε_0 is the vacuum permittivity. Therein, from calculating the plasma frequency it is possible to obtain the carrier density of a homogeneous nanoparticle. In the case of the heterogeneous nanoparticle, the expression above must take into account the different materials and their geometrical arrangement inside the spherical particle. The Maxwell-Garnett effective medium approximation can successfully be applied in order to obtain an effective dielectric constant $\varepsilon_{eff}(\omega)$ which is used in the studied case of core-shell structures.[13] The mixing formula yields:

$$\varepsilon_{eff}(\omega) = \varepsilon_{shell}\left(\frac{(\varepsilon_{core} + 2\varepsilon_{shell}) + 2F(\varepsilon_{core} - \varepsilon_{shell})}{(\varepsilon_{core} + 2\varepsilon_{shell}) - F(\varepsilon_{core} - \varepsilon_{shell})}\right) \tag{17}$$

where $\varepsilon_{eff}(\omega)$ is the effective dielectric constant, ε_{shell} is the dielectric permittivity of the shell material, ε_{core} is the dielectric permittivity of the core, $F = (R_{core}/R)^3$ is the volume ratio between the core volume and the total nanoparticle volume. Both ε_{shell} and ε_{core} are calculated with Equation (14). Equation (17) can be applied recursively in order to calculate multiple layered core-shell structures. In particular, we exploited a three-layer structure for modelling the depletion layer-shell-

core arrangement inside the studied NCs (Supplementary Figure 12). The effective dielectric constant is obtained as:

$$\varepsilon_{eff1}(\omega) = \varepsilon_{shell}\left(\frac{(\varepsilon_{core}+2\varepsilon_{shell})+2F_1(\varepsilon_{core}-\varepsilon_{shell})}{(\varepsilon_{core}+2\varepsilon_{shell})-F_1(\varepsilon_{core}-\varepsilon_{shell})}\right);$$

$$\varepsilon_{eff}(\omega) = \varepsilon_{DL}\left(\frac{(\varepsilon_{eff1}+2\varepsilon_{DL})+2F_2(\varepsilon_{eff1}-\varepsilon_{DL})}{(\varepsilon_{eff1}+2\varepsilon_{DL})-F_2(\varepsilon_{eff1}-\varepsilon_{DL})}\right) \qquad (18)$$

where $\varepsilon_{eff1}(\omega)$ is the effective dielectric constant considering only the core-shell structure without the depletion layer, $F_1 = (R_{core}/(R_{core}+t_s))^3$ is the volume ratio between the core volume and the core + shell volume, $F_2 = ((R_{core}+t_s)/R)^3$ is volume ratio between the core + shell volume and the total nanoparticle volume, ε_{DL} is the dielectric permittivity of the depletion layer. In the case of the depletion layer permittivity ε_{DL}, Equation (14) simplifies to $\varepsilon_{DL}(\omega) = \varepsilon_{\infty}$ since the plasma frequency of the depletion layer $\omega_{p,DL} = 0$ (since $n_e = 0$).

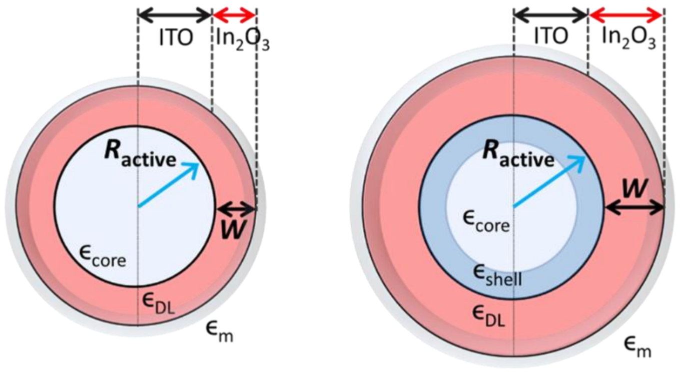

Supplementary Figure 12. **Multi-layer model for core-shell plasmonic NCs.** Schematic illustration of the multi-layer model implemented to analyse the optical response of ITO-In_2O_3 NCs. The inner part of the volume delimited with R_{active}, is assumed to be the only region in which the electrons are free to oscillate and contribute to the plasmon resonance. This active region consists of an inner uniformly doped core with dielectric constant ε_{core} and a shell with dielectric constant ε_{shell}, initially completely depleted of free electrons, responsible for a second mode of the LSPR. The two concentric spheres are surrounded by a depleted layer (with fixed ε_{DL}) of thickness W and they are immersed in a dielectric medium (fixed ε_m).

Fitting model

In order to fit the spectra, a three-layer model was implemented. While a two-layer model (structured with one active region and one superficially depleted region) is sufficient to successfully fit as-synthesized and photodoped spectra of small particles, it fails for particles with a shell thickness (t_s) larger than the critical thickness of t_s^* = 2.7 nm. This choice relies on the fact that in the case of one peak spectrum only one electronic core oscillator is needed, while when in core-shell NCs a double-peak absorption appears two oscillators are needed. The two-layer model can be seen as a particular case of the three-layer model. As a matter of fact, when the contribution from the shell is negligible a single electronic population can be considered responsible for the plasmonic response. The remaining part of the nanoparticle is just occupied by the depletion layer, which is plasmonically inactive but alters the dielectric environment that the core experiences. In contrast, the three-layer model was needed to correctly fit all samples. Some NCs developed a double peak response only after photodoping (due to the addition of extra charges in the shell), while even bigger NCs presented

the double feature even in the as-synthesised case. In this latter case, two of the three layers are plasmonically active (core and shell), while the third layer is held by the depletion region. Moreover, the observed redshifts of the main peak in the absorption spectra after photodoping require the presence of the depletion layer to be explained for both the aforementioned cases.

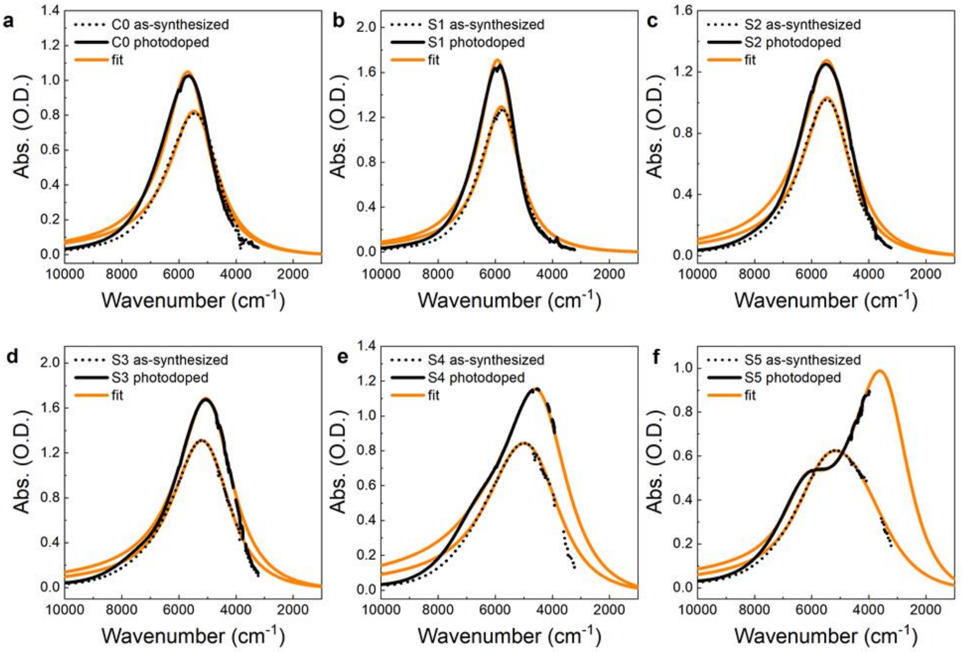

Supplementary Figure 13. **Absorbance of sample C0-S5** in the as-synthesized (dotted line) and photodoped (continue line) cases. The fitting of the experimental data using the multi-layer optical model is reported with the orange lines. Each panel is representative of a different shell thickness (t_s). **a** t_s = 0 nm; **b** t_s = 1.15 nm; **c** t_s = 1.90 nm; **d** t_s = 2.30 nm; **e** t_s = 2.90 nm; **f** t_s = 4.25 nm.

The two-layer model relies on the fit of six parameters, which are the number of electrons in the core, $n_{e,core}$, the number of electrons in the shell, $n_{e,shell}$, the core radius, R_{core}, the core damping parameter, γ_c, the shell damping parameter, γ_s, and the solution volumetric concentration, $p = N_p \cdot V_p$, where N_p, is the density of nanoparticle in the solution and V_p, is the average nanoparticle's volume. The three-layer model adds one more parameter to the previous ones: S, which is the radius of the active shell. The algorithm used for finding the best fit with both the models is the particle swarm optimization algorithm, implemented with Matlab software. This is preferable to least squares fitting function because the solution does not depend on the initial guess of the parameters but the global best fit (if exists) is found exploring all the possible combination of parameters between the lower and higher bounds. It must be noticed that the bounds for the photodoped case depends on the solution of the same as-synthetised particle. In order to have consistent solutions without overfitting, a final comparison between the solutions found for the as-synthetized case with the photodoped ones is needed. The best fit found for the as-synthesized and photodoped samples are showed in Supplementary Figure 13, and the extracted parameters are reported in Supplementary Table 2 and Supplementary Table 3 respectively.

Supplementary Table 2. **Extracted parameters for three-layer model fit for the as-synthetized samples.**

As-synthetized	C0	S1	S2	S3	S4	S5
$N_{e,core}$	703	911	912	916	914	937
$N_{e,shell}$	0	0	0	49	75	250
R_{core} (nm)	5.3	5.32	5.45	5.62	5.7	5.6
R_{active} (nm)	5.3	5.32	5.45	5.83	6.03	6.56
γ_c (cm^{-1})	$2.08 \cdot 10^3$	$1.62 \cdot 10^3$	$2.04 \cdot 10^3$	$1.88 \cdot 10^3$	$2.35 \cdot 10^3$	$1.66 \cdot 10^3$
γ_s (cm^{-1})	$10 \cdot 10^3$	$10 \cdot 10^3$	$10 \cdot 10^3$	$3.19 \cdot 10^3$	$3.62 \cdot 10^3$	$4.60 \cdot 10^3$
p	$3.40 \cdot 10^{-5}$	$5.68 \cdot 10^{-5}$	$7.87 \cdot 10^{-5}$	$11.98 \cdot 10^{-5}$	$11.73 \cdot 10^{-5}$	$11.71 \cdot 10^{-5}$
W (nm)	0.2	1.33	1.95	1.97	2.37	3.19

Supplementary Table 3. **Extracted parameters for three-layer model fit for the photodoped samples.**

Photodoped	C0	S1	S2	S3	S4	S5
$N_{e,core}$	823	1136	1171	1177	1111	528
$N_{e,shell}$	0	0	0	99	327	1365
R_{core} (nm)	5.5	5.73	6.02	6.38	6.39	4.59
R_{active} (nm)	5.5	5.73	6.02	6.62	7.26	9.45
γ_c (cm^{-1})	$1.91 \cdot 10^3$	$1.53 \cdot 10^3$	$2.12 \cdot 10^3$	$2.01 \cdot 10^3$	$2.87 \cdot 10^3$	$1.36 \cdot 10^3$
γ_s (cm^{-1})	$10 \cdot 10^3$	$10 \cdot 10^3$	$1.97 \cdot 10^3$	$5.67 \cdot 10^3$	$2.60 \cdot 10^3$	$2.58 \cdot 10^3$
p	$3.4024 \cdot 10^{-5}$	$5.68 \cdot 10^{-5}$	$7.87 \cdot 10^{-5}$	$11.98 \cdot 10^{-5}$	$11.73 \cdot 10^{-5}$	$11.71 \cdot 10^{-5}$
W (nm)	0	0.92	1.38	1.18	1.14	0.3

Quantum effects

In order to evaluate the impact of quantum effects, we analyze here deviations from the Drude model for sample C0. Sample C0 has the smallest radius and the minimum number of electrons compared to all the other samples, representing the case for which quantum confinement effects should be stronger. We calculate the quantization energy (Δ_{sp}) and compare it to the plasmon frequency (ω_{LSPR}) obtained by the classical approach.[14]

First, we calculate sample C0 bulk plasma frequency ($\omega_{p,C0}$) as follows:

$$\omega_{p,C0} = \sqrt{\frac{n_e e^2}{\varepsilon_0 m^*}} = 2.99 \cdot 10^{15}\ \text{rad s}^{-1} = 15873\ \text{cm}^{-1} \tag{19}$$

where, $n_e = 1.13 \cdot 10^{27}\text{m}^{-3}$ is the electron density obtained from the sample C0, $e = 1.602 \cdot 10^{-19}$ C, $\varepsilon_0 = 8.854 \cdot 10^{-12}$ F m^{-1} and $m^* = 0.4\, m_e = 3.644 \cdot 10^{-31}$Kg. The plasmon frequency ω_{LSPR} in the dipolar approximation of the nanocrystal is given by:

$$\omega_{LSPR} = \sqrt{\frac{\omega_{p,C0}{}^2}{\varepsilon_\infty + 2\varepsilon_m} - \Gamma^2} = 0.6379 eV = 5145\ cm^{-1} \tag{20}$$

where $\varepsilon_\infty = 4$ is the high-frequency dielectric constant of the ITO nanocrystal, $\varepsilon_m = 2.09$ is the dielectric constant of the medium (toluene) and $\Gamma = \gamma_{c,C0} = 2080\ cm^{-1}$ is the damping parameter accounting for electron-electron scattering for the sample C0 (Supplementary Table 2).

Finally, we calculate the quantization energy (Δ_{sp}) as derived in Refs.[15,16] obtaining:

$$\Delta_{sp} = \frac{\pi^2 \hbar^2}{2m^* R^2} = 33.4\ meV = 270\ cm^{-1}. \tag{21}$$

Since $\omega_{LSPR} \gg \Delta_{sp}$, we find that the classical Drude model is more suitable for describing the plasmonic response of sample C0. Quantum effects represent a minor correction, which can be neglected for this system. We conclude that this is valid also for samples with larger radius and higher electron number (given a constant n_e).

Supplementary References

1. Seiwatz, R. & Green, M. Space charge calculations for semiconductors. *J. Appl. Phys.* **29**, 1034–1040 (1958).

2. Zandi, O. *et al.* Impacts of surface depletion on the plasmonic properties of doped semiconductor nanocrystals. *Nat. Mater.* **17**, 710–717 (2018).

3. Staller, C. M. *et al.* Tuning Nanocrystal Surface Depletion by Controlling Dopant Distribution as a Route Toward Enhanced Film Conductivity. *Nano Lett.* **18**, 2870–2878 (2018).

4. Kriegel, I. *et al.* Light-Driven Permanent Charge Separation across a Hybrid Zero-Dimensional/Two-Dimensional Interface. *J. Phys. Chem. C* **124**, 8000–8007 (2020).

5. Dixon, S. C., Scanlon, D. O., Carmalt, C. J. & Parkin, I. P. n-Type doped transparent conducting binary oxides: an overview. *J. Mater. Chem. C* **4**, 6946–6961 (2016).

6. Thirumoorthi, M. & Thomas Joseph Prakash, J. Structure, optical and electrical properties of indium tin oxide ultra thin films prepared by jet nebulizer spray pyrolysis technique. *J. Asian Ceram. Soc.* **4**, 124–132 (2016).

7. Li, Y., Bu, Y., Liu, Q., Zhang, X. & Xu, J. High photocatalytic activities of zinc oxide nanotube arrays modified with tungsten trioxide nanoparticles. *Chinese J. Catal.* **39**, 54–62 (2018).

8. Liao, L. *et al.* Theoretical study on the photodoping effects in La 1- x Sr x MnO 3 /SrNb y Ti 1- y O 3 p-n heterojunction. *Phys. status solidi* **206**, 1655–1659 (2009).

9. Bohren, C. F. & Huffman, D. R. *Absorption and Scattering of Light by Small Particles*. *Absorption and Scattering of Light by Small Particles* (Wiley, 1998). doi:10.1002/9783527618156.

10. Myroshnychenko, V. *et al.* Modelling the optical response of gold nanoparticles. *Chem. Soc. Rev.* **37**, 1792–1805 (2008).

11. Lounis, S. D., Runnerstrom, E. L., Bergerud, A., Nordlund, D. & Milliron, D. J. Influence of dopant distribution on the plasmonic properties of indium tin oxide nanocrystals. *J. Am. Chem. Soc.* **136**, 7110–7116 (2014).

12. Mergel, D. & Qiao, Z. Dielectric modelling of optical spectra of thin In 2 O 3 : Sn films. *J. Phys. D. Appl. Phys.* **35**, 794–801 (2002).

13. Gibbs, S. L. *et al.* Dual-Mode Infrared Absorption by Segregating Dopants within Plasmonic Semiconductor Nanocrystals. *Nano Lett.* **20**, 7498–7505 (2020).

14. Zhang, H., Kulkarni, V., Prodan, E., Nordlander, P. & Govorov, A. O. Theory of quantum plasmon resonances in doped semiconductor nanocrystals. *J. Phys. Chem. C* **118**, 16035–16042 (2014).

15. Harrison, W. A. *Applied Quantum Mechanics*. (WORLD SCIENTIFIC, 2000). doi:10.1142/4485.

16. Flügge, S. *Practical Quantum Mechanics*. (Springer Berlin Heidelberg, 1971). doi:10.1007/978-3-642-61995-3.
